# Metabolic, inflammatory and subclinical endothelial alterations in patients with moderate-to-severe psoriasis: an investigation letter^؆^

**DOI:** 10.1016/j.abd.2026.501444

**Published:** 2026-08-07

**Authors:** Tiago Almeida Santos Costa, Isabella Bonilha de Oliveira, Sheila Tatsumi Kimura Medorima, Simone Appenzeller, Erica Ivana Lazaro Gomes, Camila Cristina Moreira Gossi, Andrei Carvalho Sposito, Renata Ferreira Magalhães

**Affiliations:** a Division of Dermatology, Faculty of Medical Sciences, Universidade Estadual de Campinas, Campinas, SP, Brazil; b Division of Cardiology, Faculty of Medical Sciences, Universidade Estadual de Campinas, Campinas, SP, Brazil; c Unit of Rheumatology, Department of Internal Medicine, Faculty of Medical Sciences, Universidade Estadual de Campinas, Campinas, SP, Brazil

Dear Editor,

Psoriasis is a chronic immune-mediated inflammatory dermatosis, currently recognized as a systemic condition associated with an increased cardiovascular risk, independently of traditional risk factors. Epidemiological studies have demonstrated a higher incidence of myocardial infarction and stroke in patients with psoriasis, particularly in moderate to severe forms.^1^, ^2^ Furthermore, the concept of the “psoriatic march” has been proposed as a consequence of the persistent systemic inflammation characteristic of this disease.^3^

The chronic inflammation associated with psoriasis may contribute to metabolic alterations, endothelial activation, and early vascular dysfunction. The integrated assessment of endothelial function through functional methods and inflammatory biomarkers has been proposed as a strategy for early detection of cardiovascular risk, although currently available findings remain heterogeneous.^4^, ^5^

In this context, the present study aimed to evaluate and compare metabolic, inflammatory, and endothelial parameters in patients with moderate to severe psoriasis without known comorbidities and in healthy age and sex-matched individuals (comparison group). Twenty-nine patients with psoriasis and twenty-one individuals in the comparison group were included. All participants underwent clinical, anthropometric, and laboratory assessments, in addition to Flow-Mediated Dilation (FMD) analysis of the brachial artery and measurement of plasma biomarkers related to systemic inflammation and endothelial activation.

Initially, an exploratory data analysis was performed using summary measures, including mean, standard deviation, median, minimum and maximum values, as well as absolute and relative frequencies. The normality of continuous variables was assessed using the Shapiro-Wilk test. For baseline comparisons between groups, continuous variables were analyzed using the Mann-Whitney test, whereas categorical variables were compared using the Chi-Square test. All analyses considered a significance level of 5% (α = 0.05).

Endothelial function was assessed by FMD, a noninvasive ultrasound-based method that measures endothelium-dependent vasodilation of the brachial artery following reactive hyperemia. This technique is used as an early marker of endothelial dysfunction and cardiovascular risk, allowing the detection of subclinical vascular alterations in chronic inflammatory diseases.^4^, ^5^

The two groups presented similar characteristics regarding age, sex, and anthropometric parameters, with no significant differences in body mass index, waist circumference, blood pressure, or heart rate. However, metabolic syndrome was significantly more prevalent in the psoriasis group. Detailed characteristics are presented in Table 1.

**Table 1:** Clinical, laboratory, and endothelial characteristics of the control and psoriasis groups.

Variable	Control	Psoriasis	p
***Parâmetros demográficos***			
Sex:			0.629^a^
Female	10 (47.6%)	17 (58.6%)	
Male	11 (52.4%)	12 (41.4%)	
Age (Years)	40.1 (10.5)	40.6 (11.7)	0.984^b^
***Anthropometric and physiological parameters***			
WHtR	56.5 (7.64)	58.7 (10.5)	0.376^b^
Abdominal circumference	95.7 (14.9)	97.8 (15.7)	0.467^b^
Waist circumference	94.7 (14.4)	98.3 (16.6)	0.316^b^
Disease duration (years)	‒	15.2 (10.5)	‒
Heart rate (bpm)	71.7 (7.78)	71.8 (12.0)	0.984^b^
BMI (kg/m²)	28.0 (5.55)	29.2 (6.94)	0.408^b^
Systolic blood pressure (mmHg)	117 (12.7)	123 (15.7)	0.246^b^
Diastolic blood pressure (mmHg)	79.1 (7.54)	78.8 (10.7)	0.768^b^
Metabolic syndrome:			**0.015**^a^
No	21 (100%)	21 (72.4%)	
Yes	0 (0.00%)	8 (27.6%)	
***Biochemical parameters***			
CKD-EPI (ml/min/1.73 m^2^)	96.1 (19.1)	107 (16.5)	**0.043**^b^
HDL cholesterol (mg/dL)	52.0 (13.2)	47.0 (10.3)	0.171^b^
Non-HDL cholesterol (mg/dL)	134 (35.8)	128 (33.5)	0.675^b^
LDL cholesterol (mg/dL)	115 (31.5)	104 (26.7)	0.244^b^
Total cholesterol (mg/dL)	186 (35.8)	173 (34.8)	0.212^b^
Creatinine (0.7–1.2 mg/dL)	0.92 (0.24)	0.79 (0.18)	0.075^b^
FIB 4	0.92 (0.45)	0.74 (0.41)	0.060^b^
Alkaline phosphatase (40–129 U/L)	53.9 (10.8)	68.2 (24.3)	**0.021**^b^
Gamma-GT (0–60 U/L)	19.6 (8.71)	24.8 (14.3)	0.207^b^
Fasting glucose (74–99 mg/dL)	76.5 (13.2)	87.6 (16.4)	**0.015**^b^
HbA1c (%)	5.50 (0.24)	5.59 (0.91)	0.517^b^
C-reactive protein (mg/L)	0.45 (0.87)	3.95 (4.85)	**<0.001**^b^
Platelets (10³/µL)	217 (55.6)	287 (72.5)	**<0.001**^b^
AST (U/L) (REF: 0‒50 U/L)	21.7 (7.15)	22.8 (20.8)	0.173^b^
ALT (U/L) (REF: 0‒50 U/L)	24.3 (15.2)	21.9 (20.2)	0.253^b^
Triglycerides (mg/dL)	86.8 (32.9)	111 (70.3)	0.311^b^
Urea (17–43 mg/dL)	30.1 (7.55)	28.2 (9.98)	0.267^b^
ESR (mm/h)	14.2 (18.0)	22.3 (17.0)	**0.034**^b^
VLDL (mg/dL)	17.3 (6.51)	22.3 (14.1)	0.316^b^
***FMD1, mm***			
0-min	0.40 (0.13)	0.35 (0.11)	0.084^b^
1-min	0.42 (0.14)	0.37 (0.12)	0.152^b^
5-min	0.42 (0.14)	0.38 (0.12)	0.173^b^
AUC	2.08 (0.68)	1.85 (0.59)	0.149^b^
% Variation	1.11 (5.10)	3.38 (6.93)	0.611^a^
***FMD2, mm (Reserve)***			
0-min	0.37 (0.06)	0.37 (0.16)	0.276^b^
1-min	0.38 (0.05)	0.39 (0.16)	0.286^b^
5-min	0.39 (0.05)	0.39 (0.16)	0.268^b^
AUC	1.92 (0.26)	1.94 (0.82)	0.255^b^
% Variation	0.59 (6.53)	2.26 (7.19)	0.753^b^
***Plasma biomarkers of endothelial function***			
Nitrate/FMD1			
0-min	21.5 (11.4)	27.1 (14.5)	0.071^b^
1-min	20.4 (9.92)	25.0 (16.9)	0.373^b^
5-min	20.8 (10.2)	20.8 (12.2)	1.000^b^
AUC	103 (48.5)	117 (67.8)	0.522^b^
Nitrite/FMD1			
0-min	0.16 (0.13)	0.29 (0.22)	**0.045**^b^
1-min	0.15 (0.17)	0.28 (0.22)	**0.041**^b^
5-min	0.15 (0.20)	0.24 (0.20)	0.066^b^
AUC	0.75 (0.86)	1.27 (1.01)	0.069^b^
Nitrate/FMD2			
0-min	19.3 (9.06)	20.9 (10.9)	0.491^b^
1-min	20.5 (9.97)	20.0 (10.3)	0.818^b^
5-min	19.3 (9.69)	19.4 (9.55)	0.783^b^
AUC	99.6 (48.1)	99.4 (49.4)	0.748^b^
Nitrite/FMD2			
0-min	0.13 (0.14)	0.33 (0.30)	**0.014**^b^
1-min	0.13 (0.15)	0.25 (0.21)	0.104^b^
5-min	0.10 (0.10)	0.27 (0.21)	**0.013**^b^
AUC	0.61 (0.61)	1.33 (0.97)	**0.017**^b^
***Inflammatory biomarkers***			
NLRP3	18.3 (12.2)	23.9 (12.9)	0.098^b^
GSDMD	37.0 (27.4)	24.5 (22.2)	**0.038**^b^
IL-1β	20.6 (52.7)	17.5 (46.3)	0.842^b^
LOX-1	238 (723)	186 (641)	0.842^b^
VCAM-1	384 (124)	528 (147)	**0.004**^b^

WHtR, Waist-to-Height Ratio; BMI, Body Mass Index; CKD-EPI, Chronic Kidney Disease Epidemiology Collaboration equation; FIB-4, Fibrosis-4 Index; CRP, C-Reactive Protein; ESR, Erythrocyte Sedimentation Rate; FMD, Flow-Mediated Dilation; AUC, Area Under the Curve; NLRP3, NOD-Like Receptor family pyrin domain containing-3; GSDMD, Gasdermin D; IL-1β, Interleukin-1 beta; LOX-1, Lectin-Like Oxidized LDL receptor-1; VCAM-1, Vascular Cell Adhesion Molecule-1, p values < 0.05 were considered statistically significant.

a Chi-Square test.

b Mann-Whitney test.

From a laboratory perspective, patients with psoriasis presented higher levels of fasting glucose, C-Reactive Protein (CRP), Erythrocyte Sedimentation rate (ESR), platelet count, and alkaline phosphatase compared with the comparison group. These findings are consistent with a systemic inflammatory state and with the association between psoriasis, low-grade inflammation, and cardiovascular risk described in the literature.^6^, ^7^

Endothelial function assessed by FMD showed no significant differences between groups, both in baseline measurements and in vasomotor reserve assessment. This result suggests that, in the absence of established cardiovascular comorbidities, measurable functional endothelial dysfunction may not be present or may not yet be detectable by vasoreactivity-based methods. This finding is consistent with the heterogeneity described in the literature, in which studies have demonstrated findings ranging from evident endothelial dysfunction to preserved vasodilatory responses in specific subgroups of patients with psoriasis.^4^, ^5^

Despite the absence of differences in FMD, alterations in nitric oxide metabolites were observed. Plasma nitrate levels did not differ between groups; however, nitrite levels were higher in patients with psoriasis at specific assessment time points. Considering that nitrite reflects, albeit indirectly, endothelial nitric oxide synthase activity and nitric oxide bioavailability, these findings may indicate early modifications in endothelial homeostasis not captured by conventional functional assessment.^8^

Additionally, increased plasma levels of VCAM-1 were observed in patients with psoriasis. VCAM-1 is involved in leukocyte adhesion to the endothelium and in the progression of atherosclerosis and has been described as a marker of endothelial activation and cardiovascular risk in chronic inflammatory diseases.^9^ Together with the nitrite-related findings, this result reinforces the hypothesis of subclinical endothelial activation in this population.

Conversely, plasma levels of Gasdermin D (GSDMD) were lower in patients with psoriasis, whereas NLRP3 and Interleukin-1 beta (IL-1β) showed no significant differences between groups. NLRP3 is a central component of the inflammasome involved in the activation of pro-inflammatory pathways, and its participation has been described in both psoriasis and vascular inflammation.^10^ The absence of differences in these markers may reflect the complexity of systemic inflammatory axis regulation, in addition to possible discrepancies between local cutaneous inflammation and circulating mediators.

Taken together, these results suggest that patients with moderate to severe psoriasis, even in the absence of known comorbidities, present metabolic and inflammatory alterations associated with markers of endothelial activation. The absence of dysfunction detectable by FMD does not exclude the presence of early cardiovascular risk-related alterations, indicating that biomarkers may complement functional endothelial assessment.^4^, ^5^, ^9^

This study has limitations, including the reduced sample size and cross-sectional design, which preclude causal inference. Furthermore, the large number of analyzed variables increases the risk of type I error due to multiple comparisons; therefore, the results should be interpreted with caution and considered exploratory.

The study was approved by the institutional ethics committee, and all participants provided written informed consent.

In conclusion, patients with moderate to severe psoriasis, even in the absence of apparent comorbidities, present metabolic and inflammatory alterations associated with markers of endothelial activation. The absence of changes in FMD suggests that functional endothelial dysfunction may not be detectable in early stages or in selected populations, whereas biomarker alterations may reflect subclinical processes. These findings reinforce the relevance of psoriasis as a systemic condition and highlight the importance of cardiovascular assessment strategies integrating functional and molecular methods.^1^, ^3^, ^6^

## ORCID ID

Isabella Bonilha de Oliveira: 0000-0002-5581-5763

Sheila Tatsumi Kimura Medorima: 0000-0002-2780-2674

Simone Appenzeller: 0000-0001-5075-4474

Erica Ivana Lazaro Gomes: 0000-0002-3178-0316

Camila Cristina Moreira Gossi: 0000-0003-4240-6925

Andrei Carvalho Sposito: 0000-0001-7127-2052

Renata Ferreira Magalhães: 0000-0001-9170-932X

## Research data availability

The entire dataset supporting the results of this study was published in this article.

## Financial support

This study was supported by the *Fundação de Amparo à Pesquisa do Estado de São Paulo* (FAPESP), grant 2023/05845-0; the *Coordenação de Aperfeiçoamento de Pessoal de Nível Superior* (CAPES), grant 001; the *Fundo de Apoio ao Ensino, à Pesquisa e Extensão, Universidade Estadual de Campinas* (FAEPEX, UNICAMP), grant 2370/22; and the *Conselho Nacional de Desenvolvimento Científico e Tecnológico* (CNPq), grant 305981/2023-4. The funding agencies had no role in the design or conduct of the study; in the collection, analysis, or interpretation of the data; or in the preparation, review, or approval of the manuscript.

## Authors’ contributions

Tiago Costa: Study conception and planning; manuscript drafting and writing; data acquisition, analysis, and interpretation.

Isabella Oliveira: Participation in the execution of exams; critical review of the manuscript.

Sheila Medorima: Participation in the execution of exams; critical review of the manuscript.

Simone Appenzeller: Critical review of the manuscript.

Érica Gomes: Technical support; Critical review of the literature.

Camila Gossi: Technical support.

Andrei Sposito: Approval of the final manuscript version; active participation in research guidance; critical review of the manuscript.

Renata Magalhães: Approval of the final manuscript version; study conception and planning; active participation in research guidance; intellectual participation; critical review of the manuscript.

## Conflicts of interest

None declared.
